# Comparison of high-intensity focused ultrasound ablation and uterine artery embolization in the management of cervical pregnancy

**DOI:** 10.3389/fmed.2022.990066

**Published:** 2022-09-16

**Authors:** Waixing Li, Xiaoli Gan, Nidhi Kashyap, Lingxiao Zou, Aiqian Zhang, Dabao Xu

**Affiliations:** ^1^Department of Obstetrics and Gynecology, The Third Xiangya Hospital, Central South University, Changsha, China; ^2^Department of Obstetrics and Gynecology, Pingxiang Maternal and Child Health Care Hospital, Pingxiang, China

**Keywords:** high-intensity focused ultrasound ablation (HIFU), uterine artery embolization (UAE), cervical pregnancy (CP), hysteroscopic curettage, clinical curative effect

## Abstract

**Background:**

Cervical pregnancy (CP) is an uncommon type of ectopic pregnancy with a rising risk to life. Currently, there is no universal protocol for the safe and effective management of CP. This study aimed to investigate the clinical efficacy of high-intensity focused ultrasound ablation (HIFU) vs. uterine artery embolization (UAE) in the management of CP to develop a standard for the treatment of CP.

**Methods:**

From January 2015 to October 2021, 36 patients with CP were diagnosed, treated, and followed up at the Department of Gynecology of Third Xiangya Hospital of Central South University. A total of 11 patients were treated with HIFU followed by suction curettage under hysteroscopic guidance, and 25 patients were treated with UAE followed by suction curettage under hysteroscopic guidance. Medical records and pregnancy outcomes were retrospectively analyzed.

**Results:**

Compared to the UAE group, the HIFU group had a shorter interval time (1.5 ± 0.21 days vs. 2.6 ± 0.26 days), shorter duration of hospitalization (5.5 ± 0.31 days vs. 6.6 ± 0.21 days), shorter recovery time of menstruation (30.6 ± 7.09 days vs. 36.9 ± 5.54 days), fewer adverse reactions (0/11 vs. 9/25), and fewer postoperative complications (1/11 vs. 8/25). There were no significant differences in age, gravidity, parity, abortion, gestational age, cardiac pulsation, admission symptoms, hemoglobin level, largest diameter of the sac/mass, serum human chorionic gonadotropin (hCG) level at admission, hospitalization expenses, hospitalization days, blood loss during curettage, degree of hCG decline, residue after curettage, fertility requirement, and pregnancy outcomes.

**Conclusion:**

Both HIFU and UAE are safe and effective in the treatment of patients with CP. Compared to UAE, HIFU treatment for CP is a safer and more effective therapeutic schedule owing to the advantages of being more minimally invasive, shorter interval time, shorter hospitalization days and recovery time of menstruation, fewer adverse reactions, and fewer postoperative complications.

## Introduction

Cervical pregnancy (CP) is a rare type of ectopic pregnancy implanted in the cervical canal, with an incidence rate of approximately 1/18,000 (1). In recent years, the incidence rate has increased rapidly owing to the widespread application of assisted reproductive technology. Patients with CP are often accompanied by gradually increasing painless vaginal bleeding, which leads to hemorrhagic shock and may be fatal without timely intervention. The diagnosis is usually based on transvaginal ultrasonography (2). Pregnancy termination is recommended once the diagnosis is confirmed. However, there is no universal protocol for the management of a CP because of the unique structure of the cervix, which is mainly composed of fibrous tissue. This is associated with a high risk of massive hemorrhage and hemorrhagic shock during uterine curettage and can lead to a hysterectomy, resulting in the loss of fertility, which seriously affects the physical and mental health of patients. Therefore, pretreatment is usually recommended before suction curettage.

Methotrexate (MTX) delivered *via* systemic or local injection is an optional drug treatment in women who are clinically stable; however, its curative effect is still uncertain. The estimated failure rate after systemic treatment is high, with a probability of hysterectomy in ~10% of cases (3–5). Uterine artery embolization (UAE) is a widely used pretreatment method to treat uncontrollable bleeding in CP and to preserve future reproductive functions. As a minimally invasive non-surgical treatment, UAE has been proven to be effective and safe in the management of CP; however, complications such as fever, abdominal pain, amenorrhea, infertility, and ovarian dysfunction may occur after UAE (6, 7). Moreover, the patient needs to immobilize the affected limb for more than 6 h after UAE, which is very uncomfortable. With the developments in medical technology, high-intensity focused ultrasound ablation (HIFU) treatment has provided a new method for the conservative management of CP. As a new non-invasive technology, HIFU has been applied in the treatment of a variety of gynecological diseases (8–13). Chen et al. (14) reported that on comparing the reproductive outcomes of patients with cesarean scar pregnancy (CSP) treated by HIFU and UAE, HIFU was more efficacious in reducing the recurrence of incision pregnancy, and similar efficacy in the treatment of CSP, with lower pain score and fewer side effects (15). Jiang et al. managed three patients with CP using HIFU combined with suction curettage under hysteroscopic guidance, showing that HIFU is feasible and effective for treating patients with CP (12).

Currently, no studies comparing the clinical efficacy of HIFU and UAE for the treatment of CP have been published. Therefore, this study aimed to preliminarily explore the clinical efficacy of HIFU and UAE in the management of CP and to follow-up on pregnancy outcomes to provide more choices for the management of CP.

## Materials and methods

The clinical data of 36 cases of CP treated with HIFU or UAE in the Department of Obstetrics and Gynecology of the Third Xiangya Hospital from January 2015 to October 2021 were retrospectively analyzed. Each patient provided informed consent, and this study was approved by the ethics committee at our institution (project no.2020-s578) on 22 September 2020. All the patients were diagnosed using transvaginal B-ultrasonography (B-US). HIFU or UAE treatment was chosen for patients after diagnosis. Among them, 11 patients were treated with HIFU followed by suction curettage under hysteroscopic guidance, and 25 patients were treated with UAE followed by suction curettage under hysteroscopic guidance.

The inclusion criteria were as follows: (1) history of amenorrhea and positive urine pregnancy test results; (2) diagnosis of CSP was confirmed by ultrasound showing a gestational sac in the cervical canal and no gestational sac in the uterine cavity; (3) gestational age <13 weeks; and (4) availability of complete clinical data.

The exclusion criteria were as follows: (1) patients with pelvic inflammatory diseases; (2) a history of trophoblastic, cardiovascular, and cerebrovascular diseases; and (3) patients with intrauterine adhesions and other diseases affecting fertility.

### The procedure of UAE

The UAE procedure has been previously described in detail (14, 15). Briefly, the patients were placed in the supine position. After local disinfection, a sheet was laid for anesthesia, the right femoral artery was catheterized by an experienced interventional radiologist using the Seldinger method, and the bilateral uterine artery was embolized with gelatin sponge granules. The angiographic examination was performed to determine whether the embolization was successful, following which the catheter along with its sheath was pulled out after successful embolization. The puncture point required manual compression for 10 min, followed by dressing with a pressure bandage, to ensure continued hemostasis. Postoperatively, patients were advised to immobilize their affected limb for more than 6 h and have bed rest for 24 h. Suction curettage under hysteroscopic guidance was performed at an average of 2.6 ± 0.26 days (range, 2–6 days) after UAE.

### The procedure of HIFU

The HIFU procedure has also been described in detail previously (12). The patients lay in the prone position on the HIFU system (JC-200 focused ultrasound tumor therapeutic system; Chongqing Haifu Medical Technology, Chongqing, China). Conscious sedation with fentanyl and midazolam *via* the peripheral vein was used during the procedure, and real-time ultrasonography was used to confirm the location of the gestational sac and monitor the response to HIFU. The ultrasound waves produced by the HIFU treatment system have an acoustic power of 400 W, they penetrate the abdominal wall and are focused on the gestational tissue. Coagulative necrosis of the targeted tissue occurs when the temperature increases. When the grayscale changed at the target tissue or when the signal of the blood flow in the gestational sac disappeared, the treatment was considered complete. Suction curettage under hysteroscopic guidance was performed at an average of 1.5 ± 0.21 days (range, 1–3 days) after HIFU treatment.

### Suction curettage under hysteroscopic guidance

The procedure of suction curettage under hysteroscopic guidance has also been described in detail previously (12), in which a hysteroscope-guided suction curettage is performed by an experienced gynecologist. After general intravenous anesthesia, the position of the gestational tissue and uterine cavity was observed using diagnostic hysteroscopy. Negative pressure suction was used to extract gestational tissue from the cervical canal. Hysteroscopy was performed to examine residual gestational tissue. The electrocoagulation method can be used to remove residual gestational tissue and stop bleeding if necessary. The specimens were then sent for pathological examination.

### Follow-up observation

Following the approved protocol, all patients were advised to return to our department for a color Doppler ultrasound examination 1 month after suction curettage. Patient parameters such as age, gestational age, gravidity, parity, admission symptoms, hemoglobin, largest diameter of the sac/mass, cardiac pulsation, serum human chorionic gonadotropin (hCG) level at admission, hospitalization expenses, hospitalization days, interval time, adverse reactions, blood loss during curettage, degree of hCG decline, residue after curettage, recovery time of menstruation, fertility requirement, complications, and pregnancy outcomes were all recorded.

Interval time refers to the time between pretreatment and hysteroscopic curettage. Adverse reactions refer to the negative symptoms reported by the patients in the interval, including lower abdominal pain, lumbago and sacrococcygeal pain, fever, right leg swelling, and increased vaginal bleeding. The degree of hCG decline (%) refers to the ratio of hCG level on the first day after curettage to that on admission. Complications refer to the secondary symptoms of patients after discharge, including infection, decreased menstruation, intrauterine adhesions, and secondary infertility.

### Statistical analysis

SPSS version 23 (IBM Company, Chicago, IL, USA) statistical analysis software was used for data analysis. Normally distributed data are presented as mean ± standard deviation, and skewed distributed data are presented as the median and interquartile range (IQR). The independent-sample *t*-test, the chi-square test, and Fisher's exact test were utilized for univariate analysis. A *p*-value of < 0.05 indicated a statistically significant difference.

## Results

The average follow-up time was 41.9 ± 21.31 months (range: 6–69 months). The average age of the patients was 33.3 ± 5.59 years (range: 26–44 years). Among the 36 patients, 6 (16.7%) patients had no symptoms, 27 (75%) patients had pure vaginal bleeding, and 3 (8.3%) patients had abdominal pain with vaginal bleeding; none of them had a history of CP. Among these patients, 12 (33.3%) patients had not given birth and 12 (33.3%) had a fertility treatment requirement. The average gestational age was 53.5 ± 17.48 days (range: 34–97 days). The average largest diameter of the sac/mass was 29.1 ± 21.27 mm (range 2.9–79 mm). We found no statistically significant differences in age, gravidity, parity, abortion, gestational age, admission symptoms, hemoglobin level, largest diameter of the sac/mass, serum hCG level, and cardiac pulsation between the two groups (*p* ≥ 0.05) (Table 1).

**Table 1:** Comparison of baseline characteristics between HIFU and UAE group.

**Variable**	**Group of HIFU (*n* = 11)**	**Group of UAE (*n* = 25)**	***P***
Mean age (years)	33.2 ± 1.75	33.3 ± 1.12	0.982
Gravidity	3.4 ± 0.73	2.8 ± 1.39	0.349
Parity	1.2 ± 0.35	0.76 ± 0.133	0.176
Abortion	2.2 ± 0.70	1.8 ± 0.26	0.529
Gestational age (D)	54.4 ± 4.77	53.2 ± 3.68	0.857
Admission symptoms	8, 72.7%	22, 88.0%	0.257
Hemoglobin (g/l)	118.6 ± 5.29	113.6 ± 4.19	0.499
Argest diameter of the sac/mass (mm)	23.2 ± 4.85	31.8 ± 4.60	0.271
Serum hCG(mIU/ml)	26863.1 ± 9662.65	24181.7 ± 5980.03	0.810
Cardiac pulsation	2, 18.2%	4, 16.0%	0.871

HIFU, high-intensity focused ultrasound ablation; UAE, uterine artery embolization.

### High-intensity focused ultrasound ablation (HIFU) ablation and UAE evaluation

We also evaluated the hospitalization conditions in the HIFU and UAE groups, including hospitalization expenses, hospitalization days, interval time, adverse reactions, intraoperative bleeding, degree of hCG decline, and residue after curettage (Table 2). We found that pretreatment with HIFU had a shorter interval and hospitalization duration than UAE. The results showed that none of the patients treated with HIFU had adverse effects during the interval time. However, 9 (9/25, 36.0%) patients treated with UAE had adverse effects, such as lower abdominal pain (5/9,55.6%), fever (1/9,11.1%), increased vaginal bleeding (1/9,11.1%), lower abdominal pain combined with fever (1/9, 11.1%), and lower abdominal pain combined with right leg swelling (1/9, 11.1%); the difference was statistically significant (*p* < 0.05). We found no statistically significant differences in hospitalization expenses, blood loss during curettage, degree of hCG decline, or residue after curettage.

**Table 2:** Comparison of resident characteristics between HIFU and UAE group.

**Variable**	**Group of HIFU (*n* = 11)**	**Group of UAE (*n* = 25)**	***P***
Hospitalization expenses (RMB)	17925.6 ± 1109.18	18499.8 ± 968.50	0.728
Hospitalization days	5.5 ± 0.31	6.6 ± 0.21	0.034*
Interval time (D)	1.5 ± 0.21	2.6 ± 0.26	0.009*
Adverse reactions	0, 0%	9, 36.0%	0.034*
Blood loss during curettage (ml)	22.6 ± 5.59	27.2 ± 5.71	0.624
Degree of hCG decline (%)	68.9 ± 4.69	72.9 ± 4.43	0.614
Residue after curettage	0, 0%	3, 12.0%	0.538

*Statistically significant difference.

HIFU, high-intensity focused ultrasound ablation; UAE, uterine artery embolization.

### Follow-up results

We followed up with 36 patients with CP. Eight patients were lost to follow-up, and the remaining 28 patients who were followed up, included 11 patients from the HIFU group and 17 patients from the UAE group. Compared with the UAE group, the HIFU group had a shorter menstrual recovery time and fewer complications. There were no significant differences in fertility requirements or pregnancy outcomes between the two groups (see Table 3).

**Table 3:** Comparison of prognosis between HIFU and UAE group.

**Variable**	**Group of HIFU (*n* = 11)**	**Group of UAE (*n* = 17)**	***P***
Recovery time of menstruation (D)	30.6 ± 7.09	36.9 ± 5.54	0.015*
Complication	1, 9.1%	8, 47.1%	0.036*
Fertility requirements	4, 36.4%	8, 47.1%	0.576
Pregnant	2, 18.2%	6, 35.3%	0.328

*Statistically significant difference.

HIFU, high-intensity focused ultrasound ablation; UAE, uterine artery embolization.

## Discussion

Patients with CP require early diagnosis and management to control bleeding as soon as possible and to avoid hysterectomy with the intention of preserving the future reproductive function of patients (1). There are several modalities available for managing a CP; however, currently, there is no standard treatment protocol. MTX has been commonly used for CP treatment but is contraindicated in patients with high levels of hCG (>5,000 mIU/ml) and/or the presence of fetal cardiac activity in the gestational sac. In addition, the efficacy of MTX is controversial, with a reported efficacy rate of ~64% and a possible hysterectomy rate of up to 10% (3, 16, 17). Therefore, MTX is perhaps not suitable for patients with CP who desire to preserve their uterus or in patients who are vitally unstable. UAE, a minimally invasive non-surgical treatment, is another commonly used method for the treatment of CP. Many published studies have confirmed that UAE is safe and effective, with the efficacy rate reaching almost 100%. Although UAE is currently the most commonly used method for managing an unstable CP, a high risk of complications and adverse effects remain a concern (18–20). With the improvement in micro-non-invasive technology, HIFU has been gradually applied in the management of various gynecological diseases (8–13). Zhu et al. (15) compared 122 cases of CSP treated with HIFU or UAE and found that compared with UAE, HIFU treatment for CSP has the advantages of a lower pain score and fewer adverse effects. Interestingly, Chen et al. (14) found that on following up on the reproductive outcomes, HIFU seemed to be superior to UAE in reducing the risk of recurrent CSP. Jiang et al. (12) reported three cases of CP, which showed that HIFU seems to be considered a conservative management for patients who desire to preserve their uterus. However, to the best of our knowledge, no study has compared the clinical efficacies of HIFU and UAE in the management of CP.

In this study, we analyzed 36 patients with CP, of whom 11 were treated with HIFU and 25 with UAE. The statistics showed no significant differences in age, gravidity, parity, abortion, gestational age, admission symptoms, hemoglobin, largest diameter of the sac/mass, serum hCG, cardiac pulsation, blood loss during curettage, degree of hCG decline (%), residue after curettage, and pregnancy outcome between the HIFU group and UAE group. Both groups of patients underwent suction curettage after the primary treatment. Therefore, HIFU was shown to be as effective as UAE.

The safety of the treatment of CP is a major concern. Many studies have reported that UAE may cause severe complications and some irreversible adverse effects, such as infection, fever, abdominal pain, infertility, and ovarian dysfunction (6, 19, 21). This is because UAE cannot precisely block the descending branch of the uterine artery. It not only blocks the blood supply to the pregnancy sac of the cervix but also reduces the blood supply to the entire uterus, fallopian tubes, and ovaries. HIFU treatment uses ultrasound generated by the treatment system to penetrate the abdominal wall and focus on the gestational tissue. These waves are converted into heat energy. When the temperature rises above 65°C, coagulation necrosis occurs, killing the targeted gestational tissue and destroying small blood vessels <2 mm around the gestational tissue (12). Therefore, HIFU may help reduce the risk of bleeding during suction curettage, without affecting the function of the remaining normal uterine tissue and ovaries. Moreover, compared with UAE, patients treated with HIFU were more comfortable in the post-operative period because they did not need to immobilize the affected limb for 6 h after treatment. We compared the adverse effects in the two groups of patients during treatment. No adverse effects were observed in patients treated with HIFU. Nine (9/25, 36.0%) patients treated with UAE experienced adverse effects. Therefore, compared with UAE, HIFU appears to be a safer and more convenient method.

The cost and prognosis of management are also key issues that need to be considered. In this study, the interval time and duration of hospitalization for the HIFU group were shorter than in the UAE group. Patients treated with HIFU had lower hospitalization expenses, although there was no significant difference in the duration and expense of hospitalization. Overall, HIFU has several advantages in terms of cost. We also followed the prognosis of these patients, including menstrual recovery, postoperative complications, and pregnancy outcomes. We found that, compared with the UAE group, the HIFU group had a shorter menstruation recovery time and fewer complications. Moreover, six patients in the UAE group and two patients in the HIFU group became pregnant again after treatment during the follow-up period; however, there was no significant difference in fertility requirements and pregnancy outcomes between the two groups. Therefore, compared to UAE, HIFU has better postoperative recovery and higher cost performance.

The limitations of the study include the retrospective study design, which may have led to some bias. Future prospective studies with a larger sample size and more accurate comparisons are needed.

## Conclusion

In summary, this study showed that both HIFU and UAE combined with hysteroscopic curettage are safe and effective in treating patients with CP. Compared with UAE, HIFU is a safer and more effective therapeutic schedule owing to its advantages of being less invasive, shorter interval time, shorter duration of hospitalization and recovery time of menstruation, fewer adverse reactions, and fewer postoperative complications. This study provides promising guidance for future standards in the management of CP.

## Data availability statement

The original contributions presented in the study are included in the article/supplementary material, further inquiries can be directed to the corresponding authors.

## Ethics statement

Written informed consent was obtained from the participants, this study was approved by the Ethics Committee at our institution (Project no.: 2020-s578).

## Author contributions

Material preparation, data collection, analysis were performed, and the first draft of the manuscript was written by WL. All authors contributed to the study's conception, commented on previous versions of the manuscript, design, read, and approved the final manuscript.

## Funding

We are grateful for the support of the Hunan Provincial Clinical Medical Technology Innovation Guiding Project (Grant No. 2020SK53605) and the Hunan Provincial Natural Science Foundation (Grant No. 2021JJ40953).

## Conflict of interest

The authors declare that the research was conducted in the absence of any commercial or financial relationships that could be construed as a potential conflict of interest.

## Publisher's note

All claims expressed in this article are solely those of the authors and do not necessarily represent those of their affiliated organizations, or those of the publisher, the editors and the reviewers. Any product that may be evaluated in this article, or claim that may be made by its manufacturer, is not guaranteed or endorsed by the publisher.
